# The influence of mammogram acquisition on the mammographic density and breast cancer association in the mayo mammography health study cohort

**DOI:** 10.1186/bcr3357

**Published:** 2012-11-15

**Authors:** Janet E Olson, Thomas A Sellers, Christopher G Scott, Beth A Schueler, Kathleen R Brandt, Daniel J Serie, Matthew R Jensen, Fang-Fang Wu, Marilyn J Morton, John J Heine, Fergus J Couch, V Shane Pankratz, Celine M Vachon

**Affiliations:** 1 Mayo Clinic College of Medicine, 200 First Street SW, Rochester, MN 55905, USA; 2 H. Lee Moffitt Cancer Center and Research Institute, 12902 Magnolia Drive, Tampa, FL 33612, USA

## Abstract

**Introduction:**

Mammographic density is a strong risk factor for breast cancer. Image acquisition technique varies across mammograms to limit radiation and produce a clinically useful image. We examined whether acquisition technique parameters at the time of mammography were associated with mammographic density and whether the acquisition parameters confounded the density and breast cancer association.

**Methods:**

We examined this question within the Mayo Mammography Health Study (MMHS) cohort, comprised of 19,924 women (51.2% of eligible) seen in the Mayo Clinic mammography screening practice from 2003 to 2006. A case-cohort design, comprising 318 incident breast cancers diagnosed through December 2009 and a random subcohort of 2,259, was used to examine potential confounding of mammogram acquisition technique parameters (x-ray tube voltage peak (kVp), milliampere-seconds (mAs), thickness and compression force) on the density and breast cancer association. The Breast Imaging Reporting and Data System four-category tissue composition measure (BI-RADS) and percent density (PD) (Cumulus program) were estimated from screen-film mammograms at time of enrollment. Spearman correlation coefficients (r) and means (standard deviations) were used to examine the relationship of density measures with acquisition parameters. Hazard ratios (HR) and C-statistics were estimated using Cox proportional hazards regression, adjusting for age, menopausal status, body mass index and postmenopausal hormones. A change in the HR of at least 15% indicated confounding.

**Results:**

Adjusted PD and BI-RADS density were associated with breast cancer (p-trends < 0.001), with a 3 to 4-fold increased risk in the extremely dense vs. fatty BI-RADS categories (HR: 3.0, 95% CI, 1.7 - 5.1) and the ≥ 25% vs. ≤ 5% PD categories (HR: 3.8, 95% CI, 2.5 - 5.9). Of the acquisition parameters, kVp was not correlated with PD (r = 0.04, p = 0.07). Although thickness (r = -0.27, p < 0.001), compression force (r = -0.16, p < 0.001), and mAs (r = -0.06, p = 0.008) were inversely correlated with PD, they did not confound the PD or BI-RADS associations with breast cancer and their inclusion did not improve discriminatory accuracy. Results were similar for associations of dense and non-dense area with breast cancer.

**Conclusions:**

We confirmed a strong association between mammographic density and breast cancer risk that was not confounded by mammogram acquisition technique.

## Introduction

Percent mammographic density represents the proportion of stromal and epithelial tissue visible on a mammogram. Mammographic density varies between women and is influenced by age, body mass index (BMI), and some epidemiologic risk factors for breast cancer such as nulliparity and late age at first birth [1,2]. Women in the highest categories of percent density (PD) are at three to five times greater risk of breast cancer relative to those in the lowest category, making it one of the strongest known risk factors for breast cancer [3,4]. The associations with breast cancer are consistent whether density is measured as a qualitative trait (for example, American College of Radiology Breast Imaging Reporting and Data System (BI-RADS) tissue composition assessment) [5,6] or a quantitative trait (for example, computer-assisted thresholding-based methods such as Cumulus) [3,7-9].

Although a substantial number of investigations of mammographic density and breast cancer have been reported [3], the majority were conducted across multiple institutions and consequently using different mammography units. This increases the potential for variation in the density estimates because of several parameters, including the influence of image acquisition. The image acquisition parameters consisting of compressed breast thickness, compression force, x-ray tube voltage peak (kVp), milliampere-seconds (mAs), and target-filter combination, where applicable, vary across mammograms to limit radiation and produce a clinically useful image. The kVp is set either automatically when using the automated exposure control (AEC) mode or by the technologist by using a reference or look-up table. The mAs value (or, equivalently, the x-ray production) is controlled by the AEC. The compression paddle setting, determined by the technologist, depends on the breast size as well as the patient's tolerance to compression force. Thus, we expect larger breasts to have larger compressed breast thicknesses. The kVp setting is a positive function of compressed breast thickness. It is well known that larger breasts are more apt to be composed of adipose tissue. Therefore, we hypothesize that both compressed breast thickness and kVp will be associated positively with non-dense area and inversely with PD. Dense breast tissue has greater x-ray attenuation properties than adipose tissue. Therefore, we expect the mAs value to have a positive correlation with dense area and also with PD. Firm or larger breasts (or both) require greater levels of compression force to both spread out and separate the breast tissue in order to maximize the image clarity. We speculate that compression force, though more difficult to connect, is associated positively with non-dense area and inversely with PD.

Given the expected associations of these acquisition parameters with mammographic density measures, we further hypothesized that the acquisition measures confound the density and breast cancer associations. But no studies known to date have directly evaluated the influence of the different acquisition parameters on the density and breast cancer association. Some studies, however, have accounted for acquisition through calibration (that is, normalizing the inter-image pixel value scale) but these findings are mixed; some show that calibration results in stronger mammographic density and breast cancer associations [10,11], whereas others have shown that calibration does not improve these associations [12,13]. In this report, we examine the association of the acquisition parameters with mammographic density measures and their influence on the density and breast cancer association within a prospective cohort study from a single large breast practice, the Mayo Mammography Health Study (MMHS) Cohort.

## Materials and methods

### Mayo Mammography Health Study eligibility

**Figure 1:**
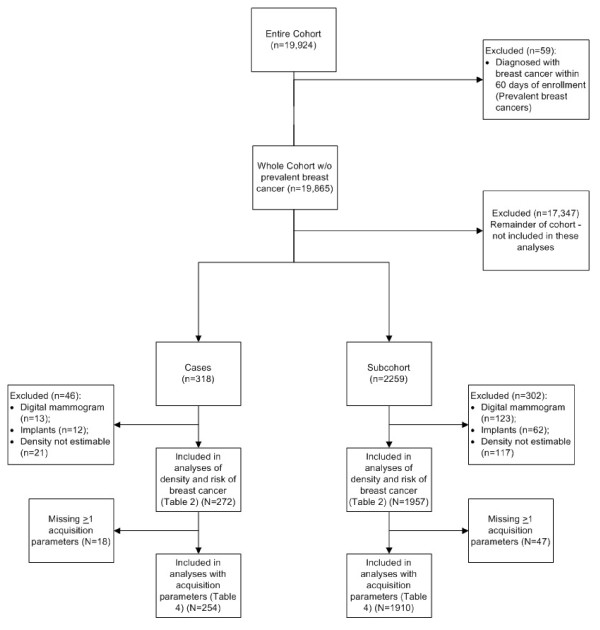
**Flow diagram of the subjects included in analyses in the Mayo Mammography Health Study**.

The MMHS prospectively enrolled patients scheduled for a screening mammogram from October 2003 through September 2006 at the Mayo Clinic in Rochester, MN. The MMHS was approved by the Mayo Institutional Review Board. Women were invited to take part if they were at least 35 years old, residents of Minnesota, Iowa, or Wisconsin (tri-state), and had no personal history of breast cancer. Women scheduled for a diagnostic mammogram (known or suspected breast cancer) were not eligible. Eligible women were mailed an invitation packet consisting of a study brochure, a consent form, a baseline questionnaire, and a permission request form to link to state tumor registries. Out of 49,032 women initially invited, 10,149 were excluded for residence outside of the tri-state area (1,698), mammogram not for screening purposes (that is, a diagnostic mammogram) (6,383), and a personal history of breast cancer (2,068). Of 38,883 eligible women, 19,924 provided written informed consented (51.2% adjusted response rate) (Figure 1). Compared with non-participants, participants were younger (11 months on average) and more likely to have ever used post-menopausal hormones (45% versus 33%), to have a first- or second-degree family history of breast cancer (19% versus 16%), more frequent mammograms (47% versus 38% had seven or more mammograms since 1986), and a history of breast biopsy (23% versus 20%) (Additional file 1).

### Mayo Mammography Health Study questionnaire

All women were asked to complete a written questionnaire that covered mammogram screening behaviors; menstrual and reproductive factors; surgeries of the breast, ovaries, and/or uterus; use of hormone therapies; medical history; family size and cancer history; use of non-steroidal anti-inflammatory medications; use of vitamins and complementary medicines; alcohol and cigarette use; physical activity; current weight and weight history; race; and education. Height and weight were also abstracted from the Mayo Clinic medical record at the medical visit closest in time to each mammogram collected for the study. To identify subjects with prevalent cancer, the medical history section of the questionnaire inquired of previous cancer diagnoses. A total of 2,283 women in the cohort reported having had at least one form of cancer (other than breast cancer) prior to enrollment. This group is excluded for analyses restricted to a 'Healthy cohort' (see Additional file 2 for a listing of prior cancer types self-reported among cohort members). Women with a prior diagnosis of breast cancer were ineligible and excluded earlier from the cohort.

### Follow-up

Follow-up for cancer occurrence was performed annually by using a combination of cancer registry data (passive follow-up) and mailed follow-up (active follow-up). All women were linked to the Mayo Clinic Tumor Registry to identify cases of cancer that had been diagnosed or treated (or both) at the Mayo Clinic since enrollment. To identify cancers external to the Mayo system, women who lived in Minnesota, Iowa, or Wisconsin and had provided written consent for linkage to external tumor registries (99.7%) were linked to their respective state tumor registries.

Active follow-up to obtain cancer and vital status was conducted in 2009 and 2010 via mail and telephone from women who had not been back to the Mayo Clinic within 12 months (thus, the medical record would not have current cancer diagnoses) and either had moved outside Minnesota, Iowa, or Wisconsin (1,755 women, 8.8%) or did not grant consent for registry linkage (62 women, 0.3%). Telephone follow-up was attempted on non-responders to the mailed contact. Thus, women who were eligible for active follow-up were contacted each year unless they were seen at the Mayo Clinic in the prior 12 months. Some women, then, could have been actively followed in one year but not the other. Active follow-up using all possible methods was successful for 83.1% in 2009 and 78.4% in 2010. By using both passive follow-up through the registries where possible and active contact by our staff, we have been able to collect cancer occurrence data on 98.8% of our cohort through 2010 (96,483 person-years).

Person-years of follow-up were computed as the amount of time since completion of the enrollment mammogram to subsequent events that differed depending upon whether the woman remained a resident of the tri-state area (and thus would be passively reported to us by the relevant state tumor registry) or moved outside. Women who resided in Minnesota, Iowa, or Wisconsin over the period were censored in the following order: (a) at the date of diagnosis with breast cancer, (b) at the date of death, or (c) on 17 December 2009. Women who moved out of these three states were censored in the following order: (a) at the date of diagnosis with breast cancer, (b) at the date of last response to cohort follow-up, (c) at the date last seen at the Mayo Clinic, or (d) at the date last known to reside in Minnesota, Iowa, or Wisconsin.

### Case-cohort design

We used the case-cohort design, in which all incident breast cancer cases that occurred in the at-risk cohort during the follow-up and a random sample of approximately 10% of those in the cohort (*n *= 2,259, plus 39 who later became cases) were selected to conduct the main analyses. We chose this design to permit prospective collection and analysis of the mammograms and risk factor data beginning at the start of the project. This design reduced the costs and time associated with obtaining mammograms and PD estimates on every woman in the cohort.

### Mammogram acquisition, retrieval, digitization, and density estimation

All mammograms at the Mayo Clinic over the time of the study were performed on one of 12 Hologic (LoRad) screen-film mammography systems (Hologic, Inc., Bedford, MA, USA) using either molybdenum (Mo)/Mo or Mo/rhodium (Rh) target-filter materials. Image acquisition parameters vary across mammograms to limit radiation and produce a clinically useful image. The compressed breast thickness (distance between the compression paddle and breast support surface) is set by the technician and is dependent upon the breast size and the patient's tolerance. In tandem, the compression force is defined by the paddle adjustment. Breast compression is used to achieve uniform breast thickness and spread the breast tissue to improve image quality. Accuracy of the measurement of thickness was within ± 5 mm; furthermore, the paddle tilt, which depends on breast size, paddle size, and compression force, showed tilt deflections of less than 1 cm when a known standard for evaluation was used. The mAs value varies due to the AEC. The AEC limits the exposure while producing a useful image and is dependent upon the breast size, breast composition, and sensor location(s). The AEC mode used for the images acquired in this study was primarily AutoFilter mode. In AutoFilter mode, the x-ray unit uses a short prepulse exposure to determine the lowest kVp selection that delivers a total exposure time of below 2 seconds or 200 mAs. In this mode, the minimum kVp selection is 25 kVp used with a Mo filter. The kVp selection rises with increased tissue attenuation up to 30 kVp, where the Mo filter is exchanged with an Rh filter. When the maximum kVp is reached (31 or 32 kVp), the mAs value may exceed 200 as needed. For very thin breasts, the AEC mode used was AutoTime, where the kVp was manually set at 23 kVp with Mo filter. KVp was tested annually with a control limit of 5.

All image acquisition parameters were manually abstracted by our staff from the printed screen-film mammogram: the compressed breast thickness (in millimeters), compression force (in pounds), x-ray tube voltage peak (kVp), milliampere-seconds (mAs), and filter. Note that when kVp is 30 or above, the filter is automatically Rh. In our data, very few individuals had a kVp of 30 or above requiring an Rh filter. Thus, the target-filter combination was limited and therefore was not considered in the analysis.

For all cases and women in the subcohort, we obtained and digitized one view from the enrollment screen-film mammogram (2003 to 2006). Screen-film mammograms were digitized on the Array 2905 laser digitizer (Array Corporation, Roden, The Netherlands), which has 50-μm (limiting) pixel spacing with 12-bit grayscale bit depth. PD was estimated by a single trained programmer (F-FW) from the craniocaudal mammogram view of the non-cancerous breast of cases and the left breast of controls. All images were scrubbed of identifying information and re-oriented so that all images were presented consistently despite the side evaluated. Thus, the reader was blinded to cancer status. Batch files were composed of both cases and controls, and a 5% repeat set of images was included within each batch file to assess reliability. Percent mammographic density (dense area divided by total area, times 100%) was estimated by the programmer by using a computer-assisted thresholding program, Cumulus [7]. Briefly, two thresholds are set by the programmer; one separates the breast from the background and the other separates dense from non-dense tissue. In the batch files examined for this study, our reader consistently demonstrated high reliability (intraclass correlation of greater than 0.93).

In addition to estimating the semi-quantitative estimation of density described above, we obtained the clinical BI-RADS four-category tissue composition assessment corresponding to the enrollment mammogram from the Mayo Clinic electronic medical record. The BI-RADS tissue composition has been routinely estimated on all screening mammograms at the Mayo Clinic since mid-1996. Mayo Clinic attending radiologists classified each mammogram into one of four categories as defined in the BI-RADS lexicon over this period (American College of Radiology, third edition): (a) the breast is almost entirely fat; (b) there are scattered fibroglandular densities; (c) the breast tissue is heterogeneously dense, which may lower the sensitivity of mammography; and (d) the breast is extremely dense, which could obscure a lesion on mammography. These ratings convey the relative possibility that a lesion may be obscured in mammography. All four mammogram views (craniocaudal and mediolateral oblique for ipsilateral and contralateral sides) contribute to the assessment of BI-RADS composition. In our study, we used the estimates that experienced radiologists assessed in the clinical setting. These radiologists did not systematically assess BI-RADS composition for this study, but this rating has shown adequate interobserver reliability [14].

### Statistical analyses

We first verified that the randomly sampled subcohort represented the full cohort by comparing basic demographic and clinical factors between the subcohort and all other cohort members (Table 1). Next, we compared these factors between breast cancer cases and the members of the subcohort by using *t *tests for continuous variables and chi-square tests for categorical variables. We estimated hazard ratios (HRs) and their 95% confidence intervals by using Cox proportional hazards regression to describe the association between the two mammographic density measures and breast cancer. Age was used as the time scale in the Cox model; age at enrollment was used as the starting point, and follow-up age was defined as age at breast cancer diagnosis for cases and age at last known follow-up for members of the subcohort. The case-cohort design was accounted for by applying sampling weights to subjects selected for the subcohort [15]. In addition to estimating the HRs, we performed tests for trend and computed the C-statistic from the Cox proportional hazards model to measure the degree to which mammographic density could discriminate risk between breast cancer cases and the other members of the subcohort. We compared the relative risk of breast cancer between groups of women classified into quartiles of PD based on values observed in the subcohort. Women in the lowest density category served as the reference group. Analyses of dense and non-dense area were conducted similarly.

**Table 1:** Demographic and risk factor distributions^a ^of the Mayo Mammography Health Study cohort (2003 to 2006)

	Analysis groups		
	**Cases, Mean or N (SD/%)^b^**	**Subcohort, Mean or N (SD/%)**	**Eligible cohort, Mean or N (SD/%)**

Number	318	2,259	19,865^c^

Age, years	61.8 (11.2)	58.0 (12.2)	58.0 (12.2)

Body mass index, kg/m^2^	28.9 (6.9)	28.1 (6.5)	28.2 (6.4)

Post-menopausal hormone use			

Current	61 (19.2%)	445 (19.4%)	3,554 (17.9%)

Former	85 (26.7%)	505 (22.0%)	4,561 (23.0%)

Never	148 (46.5%)	1,143 (49.7%)	9,986 (50.3%)

Unknown	24 (7.6%)	205 (8.9%)	1,764 (8.9%)

Post-menopausal	252 (79.3%)	1,592 (69.3%)	13,627 (68.6%)

Imaging parameters			

Number	254	1,910	

Percent density	19.1 (13.7)	17.6 (14.1)	

Dense area, mm^2^	2,622 (1,939)	2,333 (1,841)	

Non-dense area, mm^2^	13,232 (6,702)	13,460 (7,198)	

Milliampere-seconds	161.0 (49.4)	157.9 (49.5)	

X-ray tube voltage peak, kVp	26.2 (1.7)	26.1 (1.5)	

Thickness, mm	53.9 (13.3)	53.2 (12.7)	

Compression force, pounds	24.7 (6.1)	24.9 (5.6)	

^a^Reported at enrollment; ^b^39 women overlap between cases and subcohort; ^c^59 women were deleted for breast cancers occurring within the first two months after enrollment. They are included in the full cohort but excluded from analyses in this paper. SD, standard deviation.

Two additional analyses were performed to ensure the integrity of our findings. First, to ensure that prevalent cancers did not influence our results, we performed the above analyses excluding 2,283 members of the cohort who had a cancer diagnosis other than breast cancer prior to baseline enrollment. Second, we performed analyses of BI-RADS density and breast cancer within the entire cohort of 19,924 women to compare with results from the case cohort. Because the PD measure was not available on the entire cohort, we were unable to compare results for this density measure.

Data for mAs, thickness, and compression force were divided into quartiles. kVp data were not normally distributed, and 55% of values were at a standard value of 25. Thus, this variable was categorized into a three-level ordinal variable. To examine the association of acquisition parameters with mammographic density, we estimated the mean and standard deviations (SDs) of PD, dense area, and non-dense area by categories of the four acquisition parameters. We also calculated Spearman correlation coefficients between acquisition parameters and density measures. Next, we examined the degree to which inclusion of each of the acquisition parameters contributed to the breast cancer association in the presence of the measure of mammographic density, and the amount that each of these acquisition parameters individually, and all measures combined, changed the HR estimates from the original models. A change in the HR estimates of 15% or greater would provide evidence of confounding. To determine the degree to which these parameters influenced the prediction of breast cancer risk, we also computed C-statistics for the proportional hazards regression models that included the acquisition parameters as covariates. Similar analyses were conducted by using dense area and non-dense area as the endpoints.

All analyses were carried out with the SAS software system (SAS Institute Inc., Cary, NC, USA). All reported *P *values are two-sided, and comparisons were adjusted for variables found to be significantly associated with density, including age, menopausal status, post-menopausal hormone use, and BMI.

## Results

Of the 19,924 women in the MMHS cohort, 59 had a prevalent breast cancer (defined as within 60 days post-enrollment), leaving 19,865 eligible women for these analyses. Incident cases included 318 breast cancers diagnosed before 31 December 2009, and the subcohort consisted of 2,298 women randomly sampled from the entire cohort. Of the subcohort, 39 became cases and 2,259 were unaffected (Table 1 and Figure 1).

The average follow-up times from enrollment mammogram to diagnosis or last follow-up were 2.4 years (SD = 1.7) for cases and 5.0 years (SD = 0.9) for the subcohort. As shown in Table 1, the mean age of cases at enrollment was 61.8 years, which was slightly higher than among members of the cohort (58.0 years) or subcohort (58.0 years). BMI was similar in all three groups. Cases were more likely than women in either the cohort or subcohort to be post-menopausal. Cases were less likely to report having never used post-menopausal hormone therapy (46.5%) than women in either the subcohort (49.7%) or cohort (50.3%).

Table 1 also displays the means and SDs of the density estimates and the four acquisition parameters. Mean (SD) PD and dense area were higher among cases (19.1% (13.7%) and 2,622 mm^2 ^(1,939), respectively) than controls (17.6% (14.1%) and 2,333 mm^2^(1,841), respectively). Of the acquisition parameters, only mAs values showed a greater mean (SD) among cases (161.0 (49.4)) compared with controls (157.9 (49.5)).

We first confirmed the mammographic density and breast cancer association in our study. As expected, both mammographic density measures, PD and BI-RADS, were associated with future risk of breast cancer (*P *trend < 0.001) (Table 2). Women with a PD of greater than 25.1% were 3.8 (95% CI 2.5 to 5.9) times more likely to develop breast cancer during the follow-up period than women with a PD of 0% to 5.0%. Similarly, women in the highest BI-RADS category (Extremely dense) compared with the lowest BI-RADS category (Almost entirely fat) were 3.0 (95% CI 1.7 to 5.1) times as likely to develop breast cancer during the follow-up period. When only invasive breast cancers were considered (*n *= 199, data not shown), risk estimates were somewhat strengthened for the PD and breast cancer association (HR 5.1, 95% CI 3.0 to 8.4 for the highest versus lowest category of PD) but not for the BI-RADS association (HR 2.7, 95% CI 1.5 to 5.0 for the highest versus the lowest level of BI-RADS density). The two additional analyses to evaluate the possible influence of prevalent cancers and the case-cohort (versus full cohort) design on our results showed no marked difference from the main analyses (Additional file 3).

**Table 2:** Association between mammographic density estimates on the enrollment (screen-film) mammograms and risk of breast cancer in the Mayo Mammography Health Study cohort (2003 to 2009)

	Number of cases (total *n *= 272)^a^	Number of person-years (total *n *= 9,958.8)^b, c^	Multivariate adjusted hazard ratio (95% CI)^c^
Percent density			

0.0-5.0	43	2,193.8	1.00 (Ref.)

5.1-15.0	79	2,746.1	2.02 (1.37, 2.97)

15.1-25.0	59	2,386.4	2.03 (1.32, 3.11)

25.1+	91	2,632.5	3.80 (2.46, 5.86)

*P *trend			< 0.001

BI-RADS lexicon			

1: Almost entirely fat	44	2,181.6	1.00 (Ref.)

2: Scattered fibroglandular densities	111	3,904.5	1.61 (1.13, 2.29)

3: Heterogeneously dense	94	3,210.1	2.02 (1.38, 2.95)

4: Extremely dense	23	662.7	2.96 (1.73, 5.07)

*P *trend			< 0.001

Analyses adjusted for age, menopausal status, post-menopausal hormone use, and body mass index. ^a^Forty-six cases were excluded from the original 318 cases for the following reasons: digital mammogram (13), implants (12), and inability to read density (21). Percent density and BI-RADS (Breast Imaging Reporting and Data System) analyses were restricted to the same women. ^b^Three hundred two in the subcohort were excluded for the following reasons: digital mammogram (123), implants (62), and inability to read density (117). Percent density and BI-RADS analyses were restricted to the same women (272 cases and 1,957 in the subcohort).

Next, we examined the association and correlation between PD, dense area, and non-dense area and the four acquisition parameters. Table 3 shows the mean (SD) density by categories of acquisition technique. mAs, thickness, and compression were inversely associated with PD, as reflected in the mean differences and correlations (r = -0.03 (*P *= 0.60), -0.25 (*P *< 0.001), and -0.14 (*P *= 0.02), respectively, for the cases and r = -0.06 (*P *= 0.008), -0.27 (*P *< 0.001), and -0.16 (*P *< 0.001), respectively, for the subcohort members). The strongest association was seen across quartiles of thickness (mean PD of 23.3% in the lowest quartile of thickness versus 13.0% in the highest thickness quartile). kVp did not show strong evidence of an association with PD across categories. Mean dense area, however, increased across levels of kVp and mAs and decreased across levels of thickness and compression force. Non-dense area increased across levels of all four acquisition parameters, and the largest correlation was seen for thickness and non-dense area (r = 0.41, *P *< 0.001 among cases; r = 0.35, *P *< 0.001 among non-cases).

**Table 3:** Association^a ^of density estimates (percent density, dense area, and non-dense area) and categories^b ^of the four acquisition parameters

	Spearman correlation coefficient(*P *value)		Category 1		Category 2		Category 3			
			
			22-25		26-27		28-32			
	**Cases** **(*n *= 254)^c^**	**Subcohort** **(*n *= 1,910)^c^**	**Mean**	**SD**	**Mean**	**SD**	**Mean**	**SD**		

Percent density	0.09 (0.15)^d^	0.04 (0.07)^d^	17.1	13.8	17.3	14.8	19.0	14.3		

Dense area	0.28 (< 0.001)^d^	0.16 (< 0.001)^d^	2,070	1,603	2,404	1,864	2,987	2,238		

Non-dense area	0.20 (0.002)^d^	0.16 (< 0.001)^d^	12,349	6,393	14,386	7,512	15,405	8,289		

			Quartile 1		Quartile 2		Quartile 3		Quartile 4	

mAs			9-126		127-173		174-194		194-371	

			Mean	SD	Mean	SD	Mean	SD	Mean	SD

Percent density	-0.03 (0.60)	-0.06 (0.008)	18.4	14.3	18.4	14.9	17.1	13.9	16.3	13.3

Dense area	0.14 (0.02)	0.09 (< 0.001)	1,958	1,481	2,432	1,843	2,415	1,867	2,534	2,086

Non-dense area	0.24 (< 0.001)	0.25 (< 0.001)	10,906	6,034	13,412	7,284	14,214	7,091	15,404	7,571

			Quartile 1		Quartile 2		Quartile 3		Quartile 4	

Thickness, mm			6-45		46-54		55-62		62-94	

Percent density	-0.25 (< 0.001)	-0.27 (< 0.001)	23.3	16.5	17.7	13.2	15.7	12.7	13.0	11.3

Dense area	-0.01 (0.89)	-0.12 (< 0.001)	2,512	1,846	2,425	1,857	2,261	1,798	2,113	1,844

Non-dense area	0.41 (< 0.001)	0.35 (< 0.001)	10,211	6,210	13,604	7,142	14,152	6,632	16,236	7,494

			Quartile 1		Quartile 2		Quartile 3		Quartile 4	

Compression force, pounds			6.6-21		22-24		25-28		28-49	

Percent density	-0.14 (0.02)	-0.16 (< 0.001)	20.5	15.1	18	14.5	16.9	14.1	14.2	11.6

Dense area	-0.01 (0.88)	-0.02 (0.39)	2,360	1,761	2,258	1,695	2,407	1,999	2,283	1,880

Non-dense area	0.26 (< 0.001)	0.31 (< 0.001)	10,859	5,945	12,788	6,749	14,153	6,759	16,536	8,160

^a^Mean and SD of density by categories are estimated using the subcohort only. Correlation coefficients estimated on cases and subcohort separately. ^b^Quartiles were used where appropriate. The distribution of voltage peak (kVp) was highly skewed and was categorized into three levels. ^c^Owing to missing acquisition parameters, 18 cases and 47 controls that were included in the analyses depicted in Table 2 are not included here. ^d^Correlation coefficients for kVp are based on the three categories and not continuous measures. mAs, milliampere-seconds; SD, standard deviation.

The strongest correlations among acquisition parameters themselves were observed between thickness with mAs (r = 0.70 for cases and subcohort) and kVp (r = 0.72 for cases and r = 0.70 for subcohort). The smallest correlations were seen between compression and the other acquisition parameters (mAs, r = 0.10 for cases and r = 0.13 for controls; kVP, r = 0.11 for cases and r = 0.10 for controls; thickness, r = 0.001 for cases and r = 0.03 for controls). This was not entirely surprising since kVp is a function of breast thickness and mAs values are influenced by breast size (correlated with thickness) and composition.

Table 4 presents the evaluation of acquisition technique on the association between age and BMI-adjusted density and risk of breast cancer. Four parameters were evaluated singly and in combination: x-ray tube kVp, mAs, compressed breast thickness, and compression force. Inclusion of these parameters did not alter the strength of the association between age and BMI-adjusted PD and breast cancer or alter the association of BI-RADS density with breast cancer. For example, each millimeter increase in thickness was associated with only a 1.08-fold increased risk of breast cancer (95% CI 0.92 to 1.26) in the analyses of PD and breast cancer risk. The other three parameters showed similar non-statistically significant associations with breast cancer. The discriminatory capacity of the model after inclusion of any of these four parameters, as estimated by the C-statistic, was not improved for the PD-breast cancer association (0.63 or 0.64 for all models, including the model with all four parameters included) or for the BI-RADS and breast cancer association (C-statistic 0.62 for all models, including the model with all four parameters included) (Table 4).

**Table 4:** Evaluation of acquisition parameters on the association between adjusted mammographic density^a ^estimated from enrollment (screen-film) mammograms and risk of breast cancer in the Mayo Mammography Health Study cohort (2003 to 2009)

				Models with addition of acquisition parameters				
			**Models with no acquisition parameters**	**kVp - 3 levels**	**mAs**	**Thickness**	**Compression**	**All 4 acquisitionparameters**

**Percent density**	**Number of cases**	**Person-years in Subcohort**	**HR^b^(95% CI)**	**HR^b^(95% CI)**	**HR^b^(95% CI)**	**HR^b^(95% CI)**	**HR^b^(95% CI)**	**HR^b^(95% CI)**

0% to 5.0%	42	2,071.0	1.00 (Ref.)	1.00 (Ref.)	1.00 (Ref.)	1.00 (Ref.)	1.00 (Ref.)	1.00 (Ref.)

5.1% to 15.0%	76	2,678.7	1.89 (1.28, 2.80)	1.89 (1.28, 2.79)	1.88 (1.27, 2.78)	1.88 (1.27, 2.78)	1.90 (1.28, 2.80)	1.88 (1.27, 2.79)

15.1% to 25.0%	53	2,327.7	1.88 (1.22, 2.91)	1.86 (1.20, 2.88)	1.86 (1.21, 2.88)	1.88 (1.22, 2.90)	1.89 (1.22, 2.92)	1.88 (1.21, 2.92)

25.1% +	83	2,511.6	3.57 (2.30, 5.55)	3.51 (2.23, 5.48)	3.51 (2.25, 5.48)	3.60 (2.32, 5.59)	3.54 (2.28, 5.50)	3.56 (2.22, 5.69)

kVp			-	1.04 (0.87, 1.25)	-	-	-	1.01 (0.82, 1.24)

mAs			-	-	1.04 (0.89, 1.21)	-	-	1.00 (0.79, 1.26)

Thickness, mm			-	-	-	1.08 (0.92, 1.26)	-	1.06 (0.83, 1.35)

Compression, pounds			-	-	-	-	0.93 (0.82, 1.06)	0.94 (0.82, 1.07)

C-statistic			0.64	0.64	0.64	0.63	0.64	0.63

BI-RADS								

1	41	2,082.8	1.00 (Ref.)	1.00 (Ref.)	1.00 (Ref.)	1.00 (Ref.)	1.00 (Ref.)	1.00 (Ref.)

2	106	3,778.4	1.63 (1.13, 2.35)	1.62 (1.12, 2.33)	1.62 (1.12, 2.33)	1.63 (1.13, 2.35)	1.64 (1.13, 2.36)	1.61 (1.11, 2.33)

3	87	3,078.4	2.04 (1.37, 3.03)	1.98 (1.32, 2.97)	2.00 (1.34, 2.98)	2.04 (1.38, 3.04)	2.04 (1.37, 3.03)	1.96 (1.30, 2.98)

4	20	649.3	2.72 (1.53, 4.81)	2.62 (1.47, 4.68)	2.65 (1.49, 4.71)	2.78 (1.57, 4.94)	2.69 (1.52, 4.77)	2.56 (1.38, 4.75)

kVp	-		-	1.07 (0.89, 1.28)	-	-	-	1.06 (0.84, 1.33)

mAs			-	-	1.06 (0.91, 1.23)	-	-	1.05 (0.85, 1.29)

Thickness, mm			-	-	-	1.07 (0.92, 1.25)	-	0.99 (0.78, 1.27)

Compression, pounds			-	-	-	-	0.92 (0.81, 1.05)	0.92 (0.80, 1.05)

C-statistic			0.62	0.62	0.62	0.62	0.62	

^a^Analyses adjusted for age, menopausal status, post-menopausal hormone use, and body mass index. ^b^Hazard ratios (HRs) corresponding to milliampere-seconds (mAs), thickness, and compression refer to a 1 standard deviation (SD) change in the respective parameter (SD: mAs = 49.5, thickness = 12.7, compression = 5.6). HRs corresponding to voltage peak (kVp) are based on a tri-level ordinal variable (22-25, 26-27, 28-32). Owing to missing acquisition parameters, 18 cases and 47 controls that were included in the analyses depicted in Table 2 are not included here. BI-RADS, Breast Imaging Reporting and Data System; CI, confidence interval; Ref., reference value.

Similar analyses were conducted by examining the association of adjusted dense area and non-dense area with breast cancer. Like the results with PD and BI-RADS, inclusion of these four acquisition parameters, singly or in combination, did not alter the association between dense area and breast cancer or non-dense area with breast cancer (Table 5). Finally, there was no evidence of interactions between PD and acquisition parameters on breast cancer risk (mAs *P *= 0.67, kVp *P *= 0.77, thickness *P *= 0.95, and compression force *P *= 0.93).

**Table 5:** Evaluation of acquisition parameters on the association between adjusted^a ^dense area and non-dense area estimated from enrollment (screen-film) mammograms and risk of breast cancer in the Mayo Mammography Health Study cohort (2003 to 2009)

				Models with addition of acquisition parameters				
			**Models with no acquisition parameters**	**kVp - 3 levels**	**mAs**	**Thickness**	**Compression**	**All 4 acquisition parameters**

**Dense area, mm^2^**	**Number of cases**	**Person-years in subcohort**	**HR^b ^(95% CI)**	**HR^b ^(95% CI)**	**HR^b ^(95% CI)**	**HR^b ^(95% CI)**	**HR^b ^(95% CI)**	**HR^b ^(95% CI)**

0 to 985	53	2,373.0	1.00 (Ref.)	1.00 (Ref.)	1.00 (Ref.)	1.00 (Ref.)	1.00 (Ref.)	1.00 (Ref.)

985.1 to 2,023	58	2,394.0	1.43 (0.97 2.10)	1.42 (0.96, 2.09)	1.42 (0.97, 2.09)	1.42 (0.97, 2.10)	1.42 (0.97, 2.09)	1.41 (0.96, 2.07)

2,023.1 to 3,237	63	2,367.9	1.70 (1.16, 2.50)	1.68 (1.14, 2.47)	1.68 (1.14, 2.48)	1.70 (1.16, 2.50)	1.72 (1.17, 2.53)	1.68 (1.14, 2.49)

3,237.1+	80	2,454.1	2.15 (1.49, 3.12)	2.10 (1.43, 3.07)	2.11 (1.45, 3.08)	2.15 (1.49, 3.12)	2.17 (1.49, 3.14)	2.07 (1.40, 3.08)

mAs			-	1.06 (0.88, 1.27)				1.05 (0.86, 1.29)

kVp					1.05 (0.90, 1.22)			1.06 (0.85, 1.33)

mm						1.05 (0.90, 1.23)		0.97 (0.77, 1.23)

Pounds							0.91 (0.80 1.04)	0.91 (0.79, 1.04)

Non-dense area, mm^2^								

0 to 985	58	2,397.1	1.00 (Ref.)	1.00 (Ref.)	1.00 (Ref.)	1.00 (Ref.)	1.00 (Ref.)	1.00 (Ref.)

985.1 to 2,023	69	2,436.5	0.90 (0.62, 1.30)	0.89 (0.61, 1.28)	0.89 (0.61, 1.28)	0.88 (0.61, 1.28)	0.92 (0.63, 1.32)	0.94 (0.64, 1.37)

2,023.1 to 3,237	67	2,360.1	0.73 (0.49, 1.10)	0.74 (0.49, 1.11)	0.73 (0.48, 1.09)	0.72 (0.48, 1.08)	0.75 (0.50, 1.14)	0.80 (0.52, 1.22)

3,237.1+	60	2,395.4	0.53 (0.33, 0.86)	0.54 (0.33, 0.87)	0.53 (0.33, 0.86)	0.53 (0.33, 0.85)	0.55 (0.34, 0.90)	0.58 (0.36, 0.95)

mAs			-	1.13 (0.95, 1.36)				1.13 (0.93, 1.38)

kVp					1.10 (0.95, 1.28)			1.16 (0.94, 1.44)

mm						1.05 (0.90, 1.23)		0.88 (0.70, 1.11)

Pounds							0.95 (0.83, 1.08)	0.92 (0.81, 1.06)

^a^Analyses adjusted for age, menopausal status, post-menopausal hormone use, and body mass index. ^b^Hazard ratios (HRs) corresponding to milliampere-seconds (mAs), thickness, and compression refer to a 1 standard deviation (SD) change in the respective parameter (SD: mAs = 49.5, thickness = 12.7, compression = 5.6). HRs corresponding to voltage peak (kVp) are based on a tri-level ordinal variable (22-25, 26-27, 28-32). Owing to missing acquisition parameters, 18 cases and 47 controls that were included in the analyses depicted in Table 2 are not included here. CI, confidence interval; Ref., reference value.

## Discussion

Within a prospective screening cohort at a single institution, we confirmed the association between age and BMI-adjusted mammographic density and breast cancer by using a subjective clinical measure or a semi-quantitative estimate. We showed that the acquisition technique was associated with percent and area density measures. However, the mammographic density and breast cancer associations were not materially influenced by adjustment for parameters of mammogram acquisition, suggesting that the density and breast cancer association is robust, at least in the screen-film setting.

Our estimates of the association between PD and breast cancer are comparable to those of other cohort studies that used computer-assisted quantitative estimates of percent mammographic density [3,4]. Our estimate that women with greater than 25% density are at 3.8-fold increased risk of breast cancer is similar to those of 3.5 to 4.4 times in nested case-control studies using similar density categories. Because these earlier studies used mammograms from multiple institutions with variations in type of mammography machine manufacturer and processing technique whereas the MMHS used mammograms from a single institution with the same machines and protocols, this report also suggests that this variability in the PD measure has not markedly biased previous reports.

We evaluated the association of mammogram acquisition on percent and area density measures. Our findings of positive associations between compressed breast thickness, compression force, and kVp with non-dense area were expected, given the associations between these measures and breast size. Because larger breasts generally have greater adipose tissue content, they also tend to have lower percent or proportion of density compared with smaller breasts with the same amount of dense area. As such, our findings of inverse associations of PD with thickness and compression were consistent. Along this line of reasoning, we also anticipated an inverse association of kVp with PD but found no evidence for this. We noted, instead, positive associations of kVp with both dense and non-dense breast area. Although somewhat difficult to interpret, this implies that larger breasts have relatively larger amounts of both adipose and dense tissue than smaller breasts, as observed in the projection image. We also found a positive association between mAs values and dense area, as originally hypothesized, but also a positive association with non-dense area and a very small but inverse association with PD. kVp and mAs appear to influence the absolute density measures to a greater extent than the ratios, but this needs to be confirmed in other studies.

We had hypothesized that these acquisition parameters may confound the association between mammographic density measures and breast cancer risk. However, inclusion of these parameters, alone or in combination, did not influence the association between density and risk of breast cancer. Also, their inclusion did not improve the discriminatory capacity of the statistical models. Therefore, in the context of screen-film mammography and the density measures considered in this report (that is, PD, BI-RADS, dense area, and non-dense area), these acquisition parameters appear not to introduce meaningful variation reflected in the density and breast cancer associations.

The lack of confounding of the density and breast cancer association by thickness was not consistent with studies that suggest that volumetric density, which is dependent on compressed thickness, is more informative than PD and area measures [11]. Our focus on the covariate-adjusted (including BMI) density phenotypes likely explains the discrepancy. In fact, models examining PD and acquisition parameters with breast cancer that did not adjust for BMI found significant associations of all of the acquisition parameters related to thickness - that is, thickness (HR 1.20, 95% CI 1.05 to 1.38), mAs (HR 1.17, 95% CI 1.03 to 1.34), and kVp (HR 1.19, 95% CI 1.01 to 1.41) - with breast cancer. However, similar to models adjusting for BMI, the parameter estimates for the associations of density with breast cancer and the discrimination capability (C-statistics) did not materially change for models with or without the acquisition technique.

Our findings need to be considered in the context of the study design of our cohort. The mammograms included in our study were from a single institution. Therefore, the mammograms in this study are less likely to include variation from x-ray unit manufacturer, film-screen combination, and film processing conditions than would mammograms in a study that included mammograms collected from multiple institutions. This may limit the generalizability of our findings. In studies with greater variation in mammogram manufacturers and acquisition techniques, it is possible that controlling for these parameters may have a greater impact. This hypothesis needs to be tested in studies that collect mammograms from multiple sources.

We know of no other studies that have evaluated the direct influence of acquisition technique parameters on the density and breast cancer association. However, some studies have been designed to account for these parameters by using calibrated approaches [10-13]. The calibrated approach seeks to nullify uncertainties (or variation) introduced by the acquisition technique differences by producing standardized data, often with the aim of making comparisons with PD. The evaluation is indirect because the calibrated measure in the comparison is not necessarily PD and not a one-to-one comparison of the same metric such as PD derived from two data representation (that is, from calibrated and raw data). Similar to our findings, some of these studies show that the density and breast cancer association is not strengthened when accounting for the acquisition technique differences when using calibration approaches [12,13]. Because calibration is a newer approach for assessing density and the calibrated density measures are normally not the same metric as PD (or BI-RADS), it is not clear at this time whether the inclusion of the technique parameters in general is not important or whether the calibration techniques require further modifications. However, our findings reinforce and emphasize the robustness of the existing area-based percent breast density measures (that is, PD and BI-RADS), at least on digitized screen-film mammography.

Strengths of our study include the prospective nature (allowing evaluation of mammograms prior to cancer), the estimation of density by two separate methods (a semi-quantitative method and a subjective clinical measure), and the ability to systematically compare responders and non-responders in our study by using existing clinical databases. BI-RADS density did not differ substantially between participants and non-participants in the MMHS cohort, and establishing the cohort within one breast screening practice allowed us to reduce other sources of variation, including x-ray equipment (manufacturer) calibrated similarly over this period and the use of one digitizer. A limitation of our study was that BI-RADS density was estimated by numerous readers over time, but this reflects the true clinical experience and how this measure would be used in practice. Owing to a lack of variability within our population, our analyses of acquisition did not include the target-filter acquisition technique. Finally, our investigations reflect only acquisition influence on density estimates from screen-film mammograms and on mammograms from one institution only. Similar studies need to be conducted on images acquired from multiple institutions and on full-field digital mammography.

## Conclusions

Results from the MMHS cohort confirm a strong association between mammographic density and risk of breast cancer which was not materially influenced by variability in image acquisition parameters. Based upon similar risk estimates for the mammographic density/breast cancer association, our data suggest that estimation of the association between breast density and breast cancer is not improved by including acquisition parameters. Mammographic density remains a robust breast cancer risk factor that merits consideration for integration into the clinical practice to inform risk assessment and possible intervention.

## Abbreviations

AEC: automated exposure control; BI-RADS: Breast Imaging Reporting and Data System; BMI: body mass index; CI: confidence interval; HR: hazard ratio; kVp: voltage peak; mAs: milliampere-seconds; MMHS: Mayo Mammography Health Study; Mo: molybdenum; PD: percent density; Rh: rhodium; SD: standard deviation.

## Competing interests

The authors declare that they have no competing interests.

## Authors' contributions

CMV, TAS, and JEO helped to conceive of the study question, to design the MMHS cohort, and to write and finalize the paper. JJH helped to conceive of the study question and to write and finalize the paper. KRB and MJM helped to design the MMHS cohort. VSP, CGS, DJS, MRJ, and FFW performed the statistical analyses, wrote the statistical section, and provided all tables and results. FJC and BAS helped to write and finalize the paper. All authors provided input and edits on the manuscript and have read and approved the final manuscript.

## Supplementary Material

Additional file 1 **Table S1**. Comparison of participants and non-participants invited to the Mayo Mammography Health Study, 2003 to 2006. Click here for file

Additional file 2 **Table S2**. Frequencies of prior cancers (except breast cancer) among 2283 women in the Mayo Mammography Health Study Cohort. Click here for file

Additional file 3 **Table S3**. Comparison of analyses utilizing the case-cohort design versus the entire cohort* and inclusion vs. exclusion of prevalent cancers. Click here for file
