# Supplementary material for: The influence of mammogram acquisition on the mammographic density and breast cancer association in the mayo mammography health study cohort
Source: Breast Cancer Res. 2012 Nov 15;14(6):R147. doi: 10.1186/bcr3357 (PMC3701143; doi:10.1186/bcr3357)
Supplement: Additional file 1 — Table S1. Comparison of participants and non-participants invited to the Mayo Mammography Health Study, 2003 to 2006. [file bcr3357-S1.DOC]

| **Supplemental Table 1. Comparison of participants and non-participants invited to the Mayo Mammography Health Study, 2003 to 2006.** | | |
| --- | --- | --- |
|  | **Participants** | **Non-Participants^*^** |
| N | 19924 | 17445 |
| Healthy Cohort | 17641 | - |
| Prior Cancer | 2283 | - |
| Mean Age (SD) | 57.5 ± 12.2 | 58.6 ± 13.8 |
| Mean BMI (SD)  Overweight (25-30)  Obese (> 30) | 27.4 ± 6.0  6335 (32%)  5462 (27%) | 28.7 ± 6.8^**^  4917 (31%)  5677 (35%) |
| Mean Parity (SD)  Nulliparous  1-2  3-4  5+ | 2.5 ± 1.9  2708 (14%)  8028 (40%)  7234 (36)  1954 (10%) | 2.7 ± 2.0  2270 (13%)  6794 (39%)  6212 (36%)  2169 (12%) |
| Ever Postmenopausal hormone use | 8936 (45%) | 5761 (33%) |
| Biopsy History (ever) | 4577 (23%) | 3489 (20%) |
| Family History of BC in 1^st^ degree relative | 3876 (19%) | 2834 (16%) |
| Menopausal status at enrollment  Pre  Post  Missing | 5443 (28%)  13760 (72%)  721 | 4905 (30%)  11600 (70%)  940 |
| Screening mammograms since 1986  1-2  3-4  5-6  7+ | 4794 (24%)  3150 (16%)  2695 (14%)  9284 (47%) | 5706 (33%)  2943 (17%)  2220 (13%)  6574 (38%) |
| BI-RADS category1  2  3  4 | 4472 (22%)  7963 (40%)  6125 (31%)  1363 (7%) | 4206 (24%)  7245 (42%)  4958 (28%)  1033 (6%) |
| Incident breast cancer cases (12/2009) | 318 | Data not available |
| ^*^Does not include data from 1,514 non-participants who did not give general permission for researchers to view medical record data.  ^**^1441 patients with unknown BMI | | |
