# Supplementary material for: The influence of mammogram acquisition on the mammographic density and breast cancer association in the mayo mammography health study cohort
Source: Breast Cancer Res. 2012 Nov 15;14(6):R147. doi: 10.1186/bcr3357 (PMC3701143; doi:10.1186/bcr3357)
Supplement: Additional file 2 — Table S2. Frequencies of prior cancers (except breast cancer) among 2283 women in the Mayo Mammography Health Study Cohort. [file bcr3357-S2.DOC]

| **Supplemental Table 2. Frequencies of prior cancers (except breast cancer) among 2283 women in the Mayo Mammography Health Study Cohort.** | | |
| --- | --- | --- |
| **ICDO** | **Cancer Site** | **Number of cases** |
| C00-C14 | Oropharyngeal | 39 |
| C15 | Esophagus | 3 |
| C16 | Stomach | 11 |
| C18 | Colon | 230 |
| C19-C21 | Rectum and anus | 54 |
| C22 | Liver and intrahepatic bile ducts | 14 |
| C23, C24 | Gall bladder and extrahepatic bile duct | 8 |
| C25 | Pancreas | 14 |
| C33, C34 | Trachea, bronchus, and lung | 102 |
| C40 | Bone | 12 |
| C43 | Melanoma (excluding genital organs) | 343 |
| C53 | Cervix | 498 |
| C54 | Corpus, body of uterus | 324 |
| C56 | Ovary, fallopian tube, broad ligament | 205 |
| C67 | Bladder | 43 |
| C64-C66, C68 | Kidney and other unspecified urinary organs including renal pelvis, ureter, urethra | 66 |
| C71 | Brain | 34 |
| C73 | Thyroid | 186 |
| C81-C85 | Lymphoma (HL and NHL) | 166 |
| C90 | Myeloma | 15 |
| C91-C95 | Leukemia | 47 |
|  | Other | 217 |
| Note: 300 women reported more than one cancer type; thus the above numbers do not sum to 2283. Women with prior breast cancer were ineligible for inclusion in the cohort. | | |
