# Supplementary material for: The influence of mammogram acquisition on the mammographic density and breast cancer association in the mayo mammography health study cohort
Source: Breast Cancer Res. 2012 Nov 15;14(6):R147. doi: 10.1186/bcr3357 (PMC3701143; doi:10.1186/bcr3357)
Supplement: Additional file 3 — Table S3. Comparison of analyses utilizing the case-cohort design versus the entire cohort* and inclusion vs. exclusion of prevalent cancers. [file bcr3357-S3.DOC]

| **Supplemental Table 3 –** **Comparison of analyses utilizing the case-cohort design vs. the entire cohort^*^ and inclusion vs. exclusion of prevalent cancers.^**^** | | | | | | | | |
| --- | --- | --- | --- | --- | --- | --- | --- | --- |
|  |  | | Whole Cohort | | | | Case-Cohort | |
|  | # of Cases | | Person-Years | Hazards Ratios (95% Confidence Interval) **^†^** | | | Person-Years | Hazards Ratios (95% Confidence Interval)**^†^** |
| **Entire cohort (including those with baseline cancers excluding breast cancers)** | | | | | | | | |
| **BI-RADS** | | 318 | 96778.3 | | (N=19,864) | 11220.5 | | (N=2576) |
| **1** | | 51 | 22031.2 | | 1.00 (REF) | 2531.1 | | 1.00 (REF) |
| **2** | | 132 | 38596.8 | | 1.68 (1.21, 2.32) | 4498.2 | | 1.70 (1.22, 2.36) |
| **3** | | 110 | 29603.7 | | 2.37 (1.67, 3.38) | 3470.7 | | 2.41 (1.69, 3.44) |
| **4** | | 25 | 6546.5 | | 3.26 (1.94, 5.48) | 720.5 | | 3.29 (1.97, 5.51) |
| **Healthy Cohort (no baseline cancer)** | | | | | | | | |
| **BI-RADS** | | 283 | 85852.4 | (N=17,541) | | | 9850.6 | (N=2251) |
| **1** | | 46 | 19227.4 | 1.00 (REF) | | | 2150.8 | 1.00 (REF) |
| **2** | | 114 | 33957.8 | 1.60 (1.13, 2.26) | | | 3928.1 | 1.55 (1.10, 2.20) |
| **3** | | 100 | 26736.7 | 2.30 (1.59, 3.33) | | | 3120.1 | 2.30 (1.58, 3.35) |
| **4** | | 23 | 5930.5 | 3.14 (1.84, 5.37) | | | 651.6 | 3.17 (1.86, 5.42) |
| ^*^Comparison of Whole Cohort vs. Case-cohort.  ^**^Comparison of Entire Cohort vs. Healthy Cohort.  ^†^Adjusted for age, menopausal status, postmenopausal hormones, and BMI. | | | | | | | | |
